# One-Stop Transcatheter Pulmonary and Tricuspid Valve Replacement for Carcinoid Heart Disease Treatment

**DOI:** 10.1016/j.jaccas.2025.106224

**Published:** 2025-12-05

**Authors:** Nicolas Veas, Fernando J. Verdugo, Jaime Álvarez, Daniel Springmuller, Camila Bontá, Fernanda Esteves, Vinicius Esteves, Manuel Méndez

**Affiliations:** a Cardiology Unit, Clinica BUPA Santiago, Santiago, Chile; b Pediatric Cardiology Department, Pontificia Universidad Católica de Chile, Santiago, Chile; c Echocardiography Laboratory, Hospital del Salvador, Santiago, Chile; d University Hospitals Harrington Heart & Vascular Institute, Cleveland, Ohio, USA; e Department of Interventional Cardiology, Rede D'Or São Luiz, Rio de Janeiro, Brazil

**Keywords:** carcinoid heart disease, pulmonary valve insufficiency, pulmonary valve regurgitation, transcatheter valve replacement, tricuspid valve insufficiency, tricuspid valve regurgitation

## Abstract

**Background:**

Carcinoid heart disease is a complex consequence of functional neuroendocrine tumors, characterized by progressive thickening and degeneration of the right-sided valvular apparatus, leading to valvular and heart failure. Management is challenging, requiring multidisciplinary considerations regarding tumor disease management, heart failure pharmacotherapy, and heart valve replacement in individuals with acceptable performance status.

**Case Summary:**

We present a case of a middle-aged man with a history of metastatic neuroendocrine tumor receiving biweekly octreotide therapy. He presented with constitutional syndrome and right-sided heart failure signs. Cardiac imaging revealed significant tricuspid and pulmonary regurgitation. The patient was a poor surgical candidate; consequently, after multimodal cardiac imaging assessment, alternative 1-stop transcatheter pulmonary and tricuspid replacement was planned and performed.

**Discussion:**

Patients with symptomatic carcinoid heart disease require comprehensive multidisciplinary evaluation. For high-risk patients with multivalvular disease, transcatheter valve replacements provide a feasible and less invasive alternative to standard surgical replacement.

**Take-Home Messages:**

One-stop transcatheter valvular interventions are a feasible therapeutic alternative to surgical replacement in patients with symptomatic carcinoid heart disease and suitable anatomy. Multimodal cardiac imaging is crucial for preprocedural assessment and the performance of transcatheter right-sided heart valve interventions.

## Visual Summary

**Figure:**
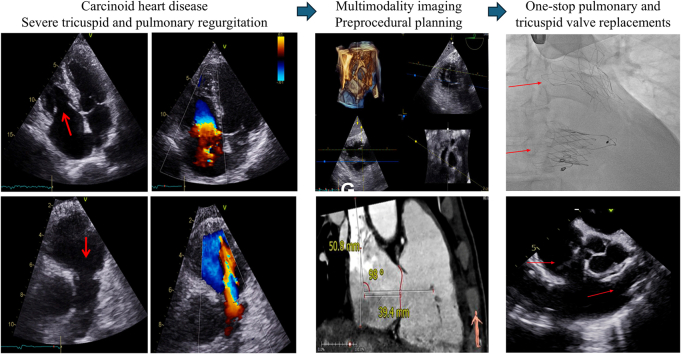

**Visual Summary:**
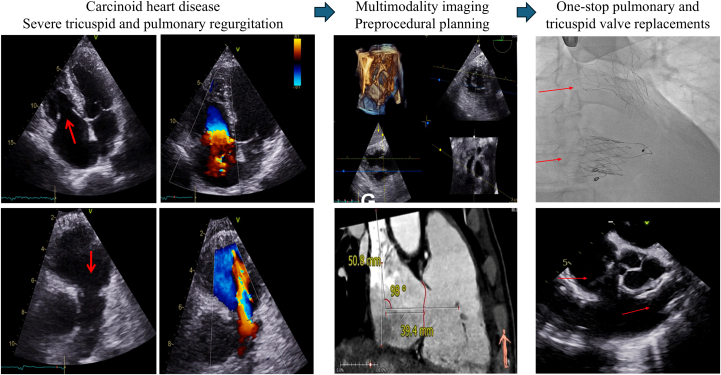
Right-Sided Carcinoid Heart Disease Diagnosis, Preprocedural Imaging and Planning, and One-Stop Transcatheter Valve Replacement Results Through Fluoroscopy and Echocardiography

Carcinoid heart disease (CHD) is a complex consequence of functional neuroendocrine tumors (NET) that secrete vasoactive amines. These hormones generate plaque-like fibrous endocardial deposits, usually on right-sided heart valves, leading to progressive thickening and degeneration of the valvular apparatus, valvular insufficiency and/or stenosis, and right-sided heart failure.^1^ Symptomatic CHD management is challenging, requiring considerations regarding heart failure pharmacotherapy, tumor disease management (somatostatin analogues, debulking, and so on), and surgical cardiac valve replacement in individuals with acceptable performance status.^1^ Isolated surgical tricuspid valve replacement is considered a high-risk procedure, with a reported perioperative mortality of 10%.^2^ Transcatheter-based interventions have shown promising results as alternative treatments for tricuspid regurgitation in population without CHD. We describe the successful transcatheter treatment of a patient with CHD.

**Take-Home Messages**

• One-stop transcatheter valvular interventions are a feasible therapeutic alternative to surgical replacement in patients with symptomatic carcinoid heart disease and suitable anatomy.
• Multimodal cardiac imaging is crucial for preprocedural assessment and the performance of transcatheter right-sided heart valve interventions.

## Case Presentation

The patient is a 53-year-old man admitted to our center for constitutional syndrome with fatigue, unintentional weight loss, chronic diarrhea, and right-sided heart failure signs (jugular distention, bilateral pleural effusions, ascites, and lower-limb edema). Cardiac auscultation revealed a holosystolic murmur (grade 2/6) at the left sternal border, which increased with inspiration.

## Past Medical History

The patient had a history of carcinoid syndrome and stage IV chronic kidney disease. Regarding the former, an ileal NET with liver metastases was diagnosed and treated with small bowel surgical resection in 2014 and hepatic debulking in 2015. ^68^Ga-DOTATATE positron emission tomography/computed tomography (PET/CT) in 2019 revealed recurrence as metastatic NET of the small bowel anastomosis, liver, and mesenteric lymph nodes, prompting treatment with octreotide 30 mg monthly, and afterward biweekly. Studies of interest before hospital stay included ^68^Ga-DOTATATE PET/CT, which showed stability of the small bowel anastomosis, hepatic implants, and mesenteric lymph nodes. Plasma chromogranin A was 3,360 ng/mL. Echocardiography revealed severe tricuspid and pulmonary regurgitation, prompting cardiology consultation. Oncologic assessment deemed the patient with an Eastern Cooperative Oncology Group/World Health Organization performance status of 1-2 and thus a candidate for second-line peptide receptor radionuclide therapy, with an expected 20-month survival rate of 65%.

## Investigations

**Figure 1:**
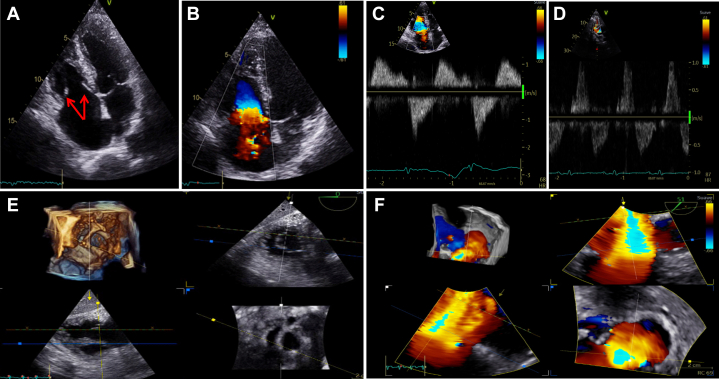
Preoperative Transthoracic and Transesophageal Echocardiography of the Tricuspid Valve (A) Transthoracic apical 4-chamber view. Red arrows indicate thickened and restricted motion of septal and mural tricuspid leaflets with a wide coaptation gap in systole. (B) Transthoracic apical 4-chamber view with color Doppler. Massive tricuspid regurgitation with a vena contracta of 14 mm. (C) Tricuspid valve continuous-wave Doppler showing no significant stenosis (mean pressure gradient 2.6 mm Hg; inflow velocity-time integral (VTI) 38 mL; pressure half-time 108 ms) and dense triangular regurgitation curve. (D) Hepatic vein flow showing systolic flow reversal. (E) Three-dimensional (3D) transesophageal echocardiographic reconstruction showing thickened and restricted motion of all tricuspid leaflets during systole. (F) 3D transesophageal reconstruction of tricuspid regurgitation. Planimetry revealed an effective regurgitant orifice area of 2.0 cm^2^ (torrential tricuspid regurgitation).

**Figure 2:**
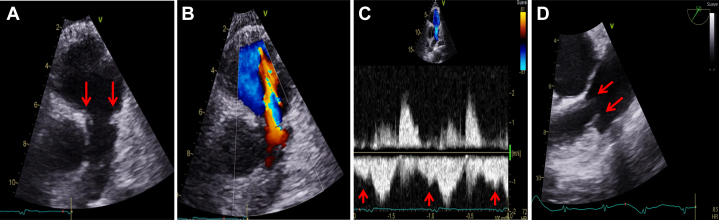
Preoperative Transthoracic and Transesophageal Echocardiography of the Pulmonary Valve (A) Transthoracic parasternal short-axis view. Red arrows indicate thickened and retracted pulmonary valve leaflets in diastole, with a wide coaptation gap. (B) Transthoracic parasternal short-axis view with color Doppler. Severe pulmonary regurgitation with jet/annulus ratio >70%. (C) Continuous-wave Doppler of the pulmonary valve. There was no significant stenosis (peak velocity 1.8 m/s; peak gradient 13 mm Hg). The pressure half-time of pulmonary regurgitation was 76 ms. The red arrow indicates end-diastolic anterograde flow at the level of the pulmonary valve, suggestive of right ventricular restrictive physiology, right ventricular dilatation, right ventricular hypertrophy, and/or long-standing pulmonary regurgitation. (D) Transesophageal echocardiography mid-esophageal short-axis zoom view of the pulmonary valve. Red arrows indicate thickened and retracted pulmonary valve leaflets in diastole.

**Figure 3:**
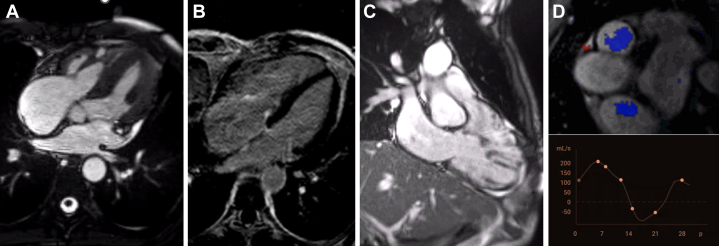
Preoperative Cardiac Magnetic Resonance (A) Four-chamber cine image with an steady-state free precession (SSFP) sequence in end-systole. Thickened tricuspid septal and mural leaflets are fixed in a partially open position with a wide coaptation defect. (B) Four-chamber view of the late gadolinium enhancement sequence in end-systole. Late gadolinium hyperenhancement of thickened septal and mural tricuspid valve leaflets. (C) Right ventricular inflow-outflow view in end-diastole. Thickened pulmonary valve leaflets with restricted motion and a wide coaptation defect. (D) Four-dimensional flow assessment at the pulmonary artery revealed a moderate pulmonary regurgitant fraction of 22% and the presence of diastolic anterograde flow, a sign associated with right ventricular restrictive physiology, right ventricular dilatation, right ventricular hypertrophy, and/or long-standing pulmonary regurgitation.

Cardiac imaging and laboratory assessments are summarized in Table 1. Transthoracic echocardiography and transesophageal echocardiography (TEE) revealed thickened and restricted pulmonary and tricuspid leaflets, with severe pulmonary and torrential tricuspid regurgitation (Figures 1 and 2, Videos 1 and 2). The right ventricle was dilated, with normal systolic function and deformation indices, and signs suggestive of restrictive physiology. There was no significant left-sided heart disease. Cardiac magnetic resonance confirmed right-chamber dilatation, normal right ventricular systolic function, signs suggestive of restrictive physiology, and significant pulmonary and tricuspid regurgitation (Figure 3, Videos 3 and 4). Right-sided heart catheterization showed mild precapillary pulmonary hypertension.

**Table 1:** Baseline Characteristics and Investigations

Baseline characteristics	
Sex	Male
Age	53 y
Dyspnea	NYHA functional class III
Body mass index	16.7 kg/m^2^
Electrocardiogram	Sinus rhythm, QRS duration 100 ms, axis +90°
Pre-existing conditions	NET for 10 y; carcinoid heart disease, stage IV chronic kidney disease, cachexia
Echocardiography	
Tricuspid regurgitation, vena contracta, EROA	Massive/torrential, 14 mm, 2.0 cm^2^
Tricuspid stenosis, peak velocity, mean gradient	Not relevant, 1.25 m/s, 2.6 mm Hg
Pulmonary regurgitation, pressure half-time	Severe, 76 ms
Pulmonary stenosis, peak velocity, peak gradient	Not relevant, 1.8 m/s, 13 mm Hg
Right ventricular end-diastolic diameter	49 mm
TAPSE, fractional area change, free wall strain	19 mm, 48%, −23%
Left ventricular end-diastolic volume	49 mL/m^2^
Left ventricular ejection fraction (3D)	60%
Cardiac magnetic resonance	
Tricuspid valve regurgitation, regurgitant fraction	Severe, 53%
Pulmonary valve regurgitation, regurgitant fraction	Moderate, 22%
Right ventricular end-diastolic volume	132 mL/m^2^
Right ventricular ejection fraction	69%
Left ventricular end-diastolic volume	69 mL/m^2^
Left ventricular ejection fraction	56%
Right-sided heart catheterization	
Pulmonary artery pressure (systolic, diastolic, mean)	37/14/22 mm Hg
Pulmonary capillary wedge pressure (mean)	9 mm Hg
Central venous pressure	15 mm Hg
Cardiac output using the Fick method	5.7 L/min
Pulmonary vascular resistance	2.3 WU
Laboratory studies	
NT-proBNP	3,198 pg/mL
Creatinine	2.63 mg/dL
Blood urea nitrogen	44 mL/dL
Albumin	2.5 g/dL
Total bilirubin	0.81 mg/dL
International normalized ratio	1.3
Hemoglobin	11.7 mg/dL
Preoperative risk assessment	
EuroSCORE II	8.9%
STS-TVS	9.8%
TRI-SCORE	7/12 (34%)

3D = 3-dimensional; EROA = effective regurgitant orifice area; EuroSCORE II = European System for Cardiac Operative Risk Evaluation II; NET = neuroendocrine tumor; NT-proBNP = N-terminal pro–B-type natriuretic peptide; STS-TVS = Society of Thoracic Surgeons Tricuspid Valve Score; TAPSE = tricuspid annular plane systolic excursion; WU = Wood Units.

## Management

**Figure 4:**
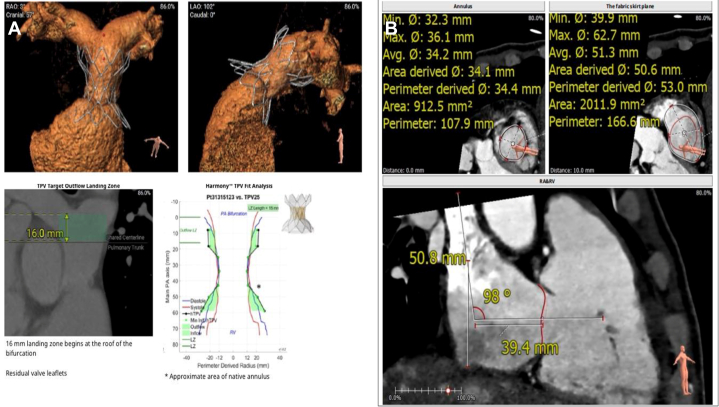
Preoperative Cardiac CT Assessment for Transcatheter Pulmonary and Tricuspid Valve Replacement Planning (A) Three-dimensional reconstruction of the computed tomography angiogram, multiplanar reformatted CT angiogram image of the RV outflow tract, and perimeter plot for Harmony valve replacement planning. (B) CT measurements for LuX-Valve planning. The tricuspid valve annulus diameter in the major axis was 52 mm; and in the short axis, 49 mm. The derived diameter from the perimeter was 50.5 mm. The derived diameter from the fabric skirt plane (10 mm above and parallel to the tricuspid annulus) was 67 mm. The angle between the tricuspid annulus and the tangent plane to the simulated anchor site was 89°. Flex assessment of the delivery system was within the specification range. CT = computed tomography; hTPV = harmony transcatheter pulmonary valve; LAO = left anterior oblique; LZ = landing zone; PA = pulmonary artery; RAO = right anterior oblique; RV = right ventricle; TPV = transcatheter pulmonary valve.

The patient received diuretic agents (furosemide, chlorthalidone, and spironolactone), intravenous albumin, and subcutaneous octreotide. The heart team considered the patient ineligible for surgery, with 34% predicted in-hospital mortality by TRI-SCORE (7/12: NYHA functional class III, right-sided heart failure signs, furosemide >125 mg daily, and kidney dysfunction), but still a candidate for transcatheter interventions given an expected oncologic survival ≥12 months. Tricuspid leaflet restrictions precluded edge-to-edge repair. CT confirmed the feasibility of transcatheter tricuspid valve replacement (TTVR) with the LuX-Valve Plus 30-40 system (Jenscare Scientific) and transcatheter pulmonary valve replacement (TPVR) with the Harmony 25 valve (Medtronic) (Figure 4).

**Figure 5:**
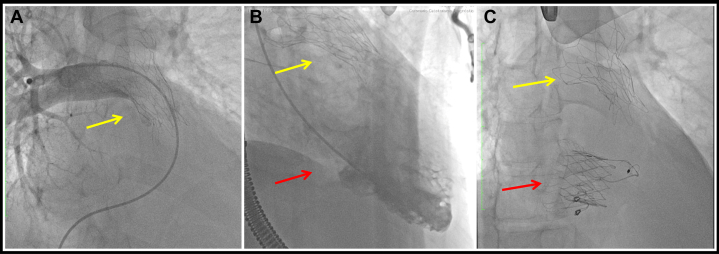
Procedural Fluoroscopy (A) Angiographic image showing implantation of a percutaneous Harmony valve (yellow arrow). (B) Image showing right ventriculography with the valve in the pulmonary position (yellow arrow) and severe tricuspid regurgitation (red arrow). (C) Angiographic image showing the LuX-Valve system in the tricuspid position (red arrow) and the Harmony valve in the pulmonary position (yellow arrow).

**Figure 6:**
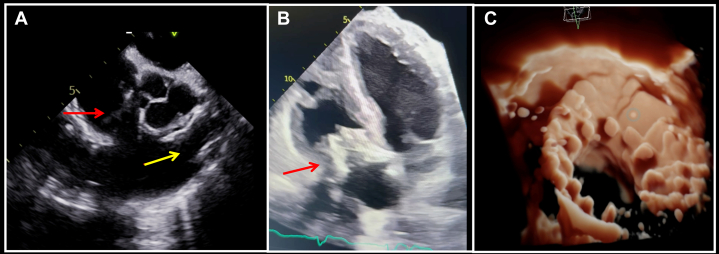
Periprocedural and Postprocedural Echocardiography (A) Transesophageal echocardiography mid-esophageal right ventricular inflow-outflow view showing the tricuspid bioprosthesis (red arrow) and pulmonary bioprosthesis (yellow arrow). (B) Transthoracic apical 4-chamber view showing the tricuspid bioprosthesis (red arrow). (C) Three-dimensional reconstruction of the tricuspid bioprosthesis.

Transcatheter interventions were performed under general anesthesia in the catheterization laboratory. TPVR was performed first, achieving successful deployment of the Harmony 25 valve (Video 5). For TTVR, right internal jugular access was obtained, and the LuX-Valve Plus #30–40 system was advanced, deployed, and anchored under TEE guidance (Videos 6 and 7). Final imaging confirmed proper valve positioning, and the absence of paravalvular leaks or pressure gradients (Figures 5 and 6, Video 8).

## Outcome and Follow-Up

The patient was admitted to the cardiac intensive care unit. Anticoagulation therapy was initiated with heparin. Subcutaneous octreotide 0.1 mg every 6 hours was administered perioperatively to prevent carcinoid crisis. Electrocardiography showed new-onset right bundle branch block. The N-terminal pro–B-type natriuretic peptide level decreased to 2,769 pg/mL. Echocardiography confirmed correct positioning of both prosthetic valves, adequate leaflet mobility, and absence of prosthetic or paravalvular regurgitation.

During the following weeks, the patient developed a systemic inflammatory response syndrome, acute kidney failure requiring hemodialysis, acute respiratory failure requiring mechanical ventilation, and hepatic failure, leading to his death 25 days after the intervention. Postoperative oncologic reassessment showed an increase in chromogranin A (26,208 ng/mL) and signs of loss of tumor differentiation on PET/CT. The unexpected changes in NET aggressiveness and frailty were considered contributing factors to his outcome.

## Discussion

Valve replacement surgery in patients with symptomatic CHD requires thorough multidisciplinary evaluation and management. Cardio-oncology guidelines recommend valve replacement surgery in patients with symptomatic CHD with severe tricuspid and/or pulmonary valve disease and an expected survival of ≥12 months.^3^ A meta-analysis of the surgical management of CHD reported high postoperative 30-day mortality rates (12%).^1^ Bioprosthetic valves are currently preferred over mechanical valves, given the increased risk of thrombosis of right-sided mechanical protheses, problematic anticoagulation in patients with high bleeding risk due to liver metastases and/or dysfunction, and plausibly decreased structural valve degeneration with novel NET therapies.^3^ In this scenario, transcatheter interventions appear to be an interesting alternative for symptomatic CHD.

No preoperative risk assessment has been validated for our patient’s case. General cardiac surgery scores, including Society of Thoracic Surgeons (STS) and EuroSCORE II (European System for Cardiac Operative Risk Evaluation II), are focused on left-sided valvular heart disease (VHD) and/or coronary interventions and underestimate the risk in tricuspid regurgitation surgery.^2^ Considering tricuspid regurgitation was our patient’s most relevant VHD, we preferred the use of TRI-SCORE, a risk score for isolated tricuspid regurgitation, which shows better prediction of in-hospital mortality in this VHD.^2^ Unfortunately, TRI-SCORE does not consider adjustments for secondary procedures such as pulmonary valve replacement (PVR), an intervention considered reasonable for our patient according to adult congenital heart disease (ACHD) guidelines.^4^

Isolated tricuspid valve replacement vs combined replacement was discussed by the heart team. Current VHD guidelines do not provide recommendations for this clinical scenario and instead suggest extrapolating from ACHD guidelines, which state that PVR is reasonable for preservation of ventricular size and function in asymptomatic patients with ventricular enlargement or dysfunction.^4^ Our patient exhibited severely dilated right ventricular end-diastolic volume (RVEDV), possible overestimation of right ventricular ejection fraction due to severe tricuspid regurgitation, and signs suggestive of restrictive right ventricular physiology. The guideline cutoff for intervention includes RVEDV ≥160 mL/m^2^; nonetheless, patients with RVEDV <150 mL/m^2^ could benefit from PVR.^5^^,^^6^ Frigiola et al^5^ reported a PVR approach in patients with ACHD and mean RVEDV of 143 mL/m^2^, resulting in normalization of right ventricular volumes and improvement in ventricular function and exercise capacity. The COMPASSION (COngenital Multicenter trial of Pulmonic vAlve regurgitation Studying the SAPIEN interventional THV) trial, comprising patients with ACHD undergoing TPVR with a mean RVEDV of 131 mL/m^2^ (N = 36), showed normalization of RVEDV and improved NYHA functional class at follow-up.^6^ An increase in right ventricular afterload after isolated TTVR was considered potentially deleterious, given the possible restrictive physiology. PVR has been reported to diminish the presence of restrictive physiology in some patients with ACHD.^7^ Furthermore, uncertainties existed regarding difficulties in performing TPVR after TTVR with the LuX-Valve Plus system. Given these factors, the 1-stop TPVR and TTVR approach was favored.

Evidence of transcatheter interventions in the population with CHD is limited. For pulmonary valve disease due to CHD, positive outcomes with TPVR have been reported using ballon-expandable valves (Melody, Medtronic, and SAPIEN, Edwards) and with a self-expanding Venus P-Valve (Venus Medtech) in patients with pulmonary artery dilatation.^1^^,^^8^

Tricuspid valve interventions have been infrequently reported in patients with CHD, which is unsurprising considering restricted leaflet motion and absence of coaptation due to a large tricuspid annulus represent unfavorable anatomy for edge-to-edge repair. Overlooking these anatomical concerns, heterotopic TTVR has been performed in patients with CHD with isolated inferior vena cava protheses using Edwards SAPIEN or bicaval TricValve (Products & Features), with mixed results.^1^ Prospective trials of TricValve outside population with CHD showed 97% technical success and significant functional status improvement in >90% of treated patients at 1-year follow-up.^9^ Although concerns regarding valvular dislodgment and large tricuspid annulus have limited orthotopic TTVR, current devices have novel anchoring mechanisms and are less limited by annular dilatation.^9^ Among these, EVOQUE (Edwards) and LuX-Valve Plus have reported procedural success rates >90%, 30-day overall mortality rates <6%, and clinical status improvement in the population without CHD.^9^ One prior successful orthotopic TTVR has been reported in a patient with CHD.^8^ In our case, aiming to maintain cardiac function physiologically and minimize hepatic congestion, orthotopic TTVR was believed to be more beneficial than heterotopic TTVR. The LuX-Valve Plus system was feasible because: 1) TEE provided high-quality imaging required for intraoperative guidance and confirmed adequate anterior and posterior tricuspid leaflet anatomy for capture by the device graspers; and 2) CT confirmed adequate jugular vein access, tricuspid annulus measurement compatible with an available device size, and optimal deployment projections. Other orthotopic TTVR devices were not available in our country. Unknowingly at the time of procedure, a case report of successful simultaneous TPVR and TTVR using Venus P-Valve and EVOQUE in a patient with CHD was under review and has recently been published.^10^

## Conclusions

For carcinoid disease in high-surgical-risk patients, transcatheter options may be reasonable in anatomically suitable patients. One-stop TPVR and TTVR seem feasible in selected patients with CHD. Multimodal imaging was crucial for preprocedural assessment and performance. To our knowledge, this is the first report of Harmony transcatheter pulmonary valve and LuX-Valve Plus systems in patients with CHD. Registry studies addressing preoperative assessment and interventional management in population with CHD are required.

## Funding Support and Author Disclosures

This work was financially supported by Clinica Bupa Santiago. The patient was not part of a clinical trial for new devices. Drs V Esteves and F Esteves were proctors for Jenscare Scientific. All other authors have reported that they have no relationships relevant to the contents of this paper to disclose.

## Appendix

Video 1 Transthoracic Echocardiography of Carcinoid Tricuspid Valve Disease Parasternal right ventricular inflow and 4-chamber views showing the tricuspid valve with thickened leaflets with restricted motion and a wide coaptation defect. The last transition shows a 3D transthoracic multiplanar reconstruction of the carcinoid tricuspid valve disease.

Video 2 Transesophageal Echocardiography of Carcinoid Pulmonary Valve Disease Mid-esophageal right ventricular outflow zoom view showing thickened pulmonary leaflets with restricted motion and a wide coaptation defect. Color Doppler shows significant pulmonary regurgitation.

Video 3 Cardiac Magnetic Resonance 4-Chamber Cine Image and Late Gadolinium Enhancement Four-chamber SSFP cine images showing normal right ventricular systolic function and thickened tricuspid septal and mural leaflets, fixed in a partially open position with a wide coaptation defect. Four-chamber view with the late gadolinium enhancement sequence in end-systole shows late gadolinium hyperenhancement of the thickened septal and mural tricuspid valve leaflets.

Video 4 Cardiac Magnetic Resonance Right Ventricular Inflow-Outflow Right ventricular inflow-outflow cine images showing thickened right valve leaflets with restricted motion and a wide coaptation defect.

Video 5 Transcatheter Pulmonary Valve Replacement Fluoroscopic cine images showing different steps of transcatheter pulmonary valve replacement with the Harmony valve. Cine images include device advancement, positioning, and result after deployment. Significant pulmonary regurgitation can be observed during angiography performed for positioning.

Video 6 Transcatheter Tricuspid Valve Replacement (A) Right ventriculography showing normal right ventricular systolic function and severe mitral regurgitation. (B) The transition shows fluoroscopy and mid-esophageal 3D transesophageal echocardiography guidance and steering of the delivery system through the center of the tricuspid valve annulus into the right ventricle. (C) Grasper exposure. (D) Grasper adjustments of the anterior and posterior tricuspid valve leaflets under mid-esophageal transesophageal echocardiography guidance.

Video 7 Transcatheter Tricuspid Valve Replacement (A) Fluoroscopy showing further valve unsheathing. (B) Transesophageal echocardiography with and without color Doppler used to identify the location of periprosthetic valvular leak and guide fine adjustments. (C) Advancement of the septal anchoring system. (D) Deployment of the septal anchoring system. (E) Prothesis liberation from the delivery system. (F) Final fluoroscopy.

Video 8 Postimplant Transesophageal and Transthoracic Echocardiography Cine Images Transesophageal echocardiography 3D reconstruction of the LuX-Valve Plus system from the surgeon’s view. The first transition shows the transesophageal echocardiography mid-esophageal right ventricular inflow-outflow view, depicting the LuX-Valve Plus and Harmony valves in situ. The second transition shows the transthoracic echocardiography biplanar view of LuX-Valve Plus. The third transition shows the apical 4-chamber view with color Doppler, depicting no evident prosthetic or periprosthetic regurgitation. The fourth transition shows the transthoracic echocardiography parasternal short-axis view, depicting the Harmony valve in situ. The fifth transition shows color Doppler of the Harmony valve, depicting no evident prosthetic or periprosthetic regurgitation.
